# Mayotte, une île enfin exempte de paludisme?

**DOI:** 10.48327/mtsi.v3i1.2023.289

**Published:** 2023-02-15

**Authors:** Jean-François Lepère, Louis Collet, Ambdoul-Bar Idaroussi, Bruno Pradines

**Affiliations:** 1 Centre médical de référence de Dzoumogné, Centre hospitalier de Mayotte, BP 04, 97600 Mamoudzou, France; 2 Laboratoire de biologie médicale, Centre hospitalier de Mayotte, BP 04, 97600 Mamoudzou, France; 3 Service de lutte antivectorielle, ARS de Mayotte, Centre Kinga, 97600 Mamoudzou, France; 4 Unité Parasitologie et entomologie, Département Microbiologie et maladies infectieuses, Institut de recherche biomédicale des armées, 19-21 bd Jean Moulin, 13005 Marseille, France; 5 Université Aix-Marseille, IRD, SSA, VITROME, 19-21 bd Jean Moulin, 13005 Marseille, France; 6 IHU Méditerranée Infection, 19-21 bd Jean Moulin, 13005 Marseille, France; 7 Centre national de référence du paludisme, 19-21 bd Jean Moulin, 13005 Marseille, France

**Keywords:** Paludisme, Élimination, *Plasmodium falciparum*, Mayotte, Comores, Océan Indien, Malaria, Elimination, *Plasmodium falciparum*, Mayotte, Comoros, Indian Ocean

## Abstract

Mayotte, département français d'outre-mer, est l'une des 4 îles de l'archipel des Comores dans l'océan Indien, situé entre Madagascar et la côte orientale de l'Afrique. Le paludisme, principalement dû à *Plasmodium falciparum*, est endémique dans l'archipel et a longtemps représenté un problème majeur de santé publique à Mayotte. Pour lutter, voire éliminer, la maladie, diverses mesures sont mises en place à Mayotte à partir de 2001. Les moyens diagnostiques et thérapeutiques ont été améliorés, la surveillance épidémiologique mise en place et la lutte antivectorielle renforcée.

De 2002 à 2021, 4 819 cas acquis localement ont été déclarés à Mayotte où l'incidence annuelle est passée de 10,3 ‰ en 2002 (1649 cas) à moins de 0,01 ‰ en 2020 (2 cas). L'incidence est inférieure à 1 ‰ depuis 2009. En 2013, l'OMS classe Mayotte parmi les territoires en phase d’élimination du paludisme. En 2021, aucun cas de paludisme acquis localement n'a été signalé sur l’île. Durant cette période 2002-2021, 1898 cas importés ont été observés. Ils proviennent principalement de l'Union des Comores (85,8%), puis de Madagascar (8,6%) et d'Afrique subsaharienne (5,6%). Depuis 2017, le nombre annuel de cas acquis localement est inférieur à 10 et décroît régulièrement (9 cas en 2017, 5 en 2018, 4 en 2019 et 2 en 2020). La distribution, à la fois dans le temps et dans l'espace, de ces rares cas acquis localement suggère que ce sont des cas introduits et non indigènes. L’étude du profil génotypique des souches plasmodiales de ces cas observés de 2017 à 2020 (17 cas analysés sur les 20 diagnostiqués) confirme qu'il s'agit certainement de cas introduits liés à des cas importés des Comores voisines.

La transmission indigène du paludisme semble être interrompue à Mayotte, mais l’île reste sous la menace d'une réintroduction via des cas importés des pays voisins. Il est temps d’élaborer un plan local de prévention de la réintroduction et de mettre en œuvre une politique volontariste de coopération régionale en matière de lutte contre le paludisme.

## Abstract (en)

Mayotte is a French overseas department and one of the 4 islands of the Comoros archipelago in the Indian Ocean, located between Madagascar and the eastern coast of Africa. Malaria, mainly by *Plasmodium falciparum*, is endemic to the archipelago and remained a major public health problem until recent years. To control and then eliminate the disease, major strategies have been established in Mayotte since 2001. Preventive measures, diagnostic testing, treatment and disease surveillance were introduced or improved.

From 2002 to 2021, 4819 autochthonous cases were reported in Mayotte where the annual incidence decreased from 10.3 per 1,000 population in 2002 (1649 cases) to less than 0.01 per 1,000 population in 2020 (2 cases). The incidence has been below 1 per 1,000 population since 2009. In 2013, WHO classified Mayotte as a territory in malaria elimination phase. In 2021, no locally acquired malaria cases were reported on the island. During this period 2002-2021, 1898 imported cases were observed. They mainly came from the Union of Comoros (85.8%), Madagascar (8.6%) and sub-Saharan Africa (5,6%). Since 2017, the annual number of locally acquired cases was less than 10 and decreased steadily (9 cases in 2017, 5 in 2018, 4 in 2019 and 2 in 2020). The distribution of these rare locally acquired cases both in time and space suggests that they were introduced and not indigenous cases. A study of the genotypic profile of the plasmodial strains of these cases observed from 2017 to 2020 (17 cases analysed out of 20 diagnosed) confirms that these were certainly introduced cases related to imported cases from the neighboring Comoros.

Malaria indigenous transmission seems to be eliminated in Mayotte, but the island remains under threat of reintroduction via cases imported from neighbouring countries. It is time to develop a local plan to prevent reintroduction and to implement a proactive policy of regional cooperation in the fight against malaria.

Mayotte, département français de l'océan Indien, est l'une des 4 îles de l'archipel des Comores situé entre Madagascar et la côte de l'Afrique de l'Est. Avec 290 000 habitants en 2021 sur un territoire de 376 km^2^, l’île est densément peuplée. Le climat y est de type tropical humide avec une saison des pluies de novembre à avril et une saison sèche de mai à octobre.

Mayotte est une zone de transmission permanente du paludisme, quasi exclusivement à *Plasmodium falciparum.* De 2012 à 2021, *P. falciparum* représente 95% des cas de paludisme observés sur l’île, suivi par *P. malariae* (4%), *P. vivax* (0,5%) et *P. ovale* (0,5%). Les deux principaux vecteurs sont *Anopheles gambiae* et *Anopheles funestus.*

Le premier programme de lutte contre le paludisme est mis en place à Mayotte en 1976. Il associe une campagne de pulvérisations systématiques d'insecticide à effet rémanent à l'intérieur des habitations, une chimioprophylaxie de masse par chloroquine (CQ) chez les femmes enceintes et les enfants et l'administration d'un traitement présomptif par CQ à tout accès fébrile. Ce programme est rapidement efficace. L'indice plasmodique global passe de 36% en 1972 à moins de 1% en 1980. À la fin des années 1980, le nombre annuel de cas est inférieur à 100 et l'incidence annuelle inférieure à 1 ‰. Le paludisme n'est alors plus considéré comme un problème de santé publique.

Malheureusement la situation se détériore rapidement au cours de la décennie suivante. À la fin des années 1990, le nombre annuel de cas est en moyenne de 1 500 et l'incidence supérieure à 10 ‰. En 2001 on enregistre le nombre record de 10 décès dans l'année [1]. Cette dégradation de la situation est principalement due à une résistance croissante à la chloroquine. En 2001, plus de 90% des souches plasmodiales testées présentent la mutation *Pfcrt* K76T associée à une résistance à la chloroquine [6].

En réponse, les autorités sanitaires de l’île mettent en place fin 2001 plusieurs mesures successives de renforcement de la lutte antipaludique selon les préconisations de l'Organisation mondiale de la Santé (OMS) [2, 3, 5].

Les moyens diagnostiques sont renforcés avec la mise à disposition de tests de diagnostic rapide (TDR) dans toutes les structures de santé de l’île. Chaque TDR positif est confirmé par la réalisation d'un frottis et d'une goutte épaisse réalisés au laboratoire du Centre hospitalier de Mayotte (CHM). L'usage des TDR met *de facto* fin à la pratique du traitement présomptif. Dorénavant, seuls les accès palustres reçoivent un traitement antipaludique. Depuis 2020, le laboratoire du CHM est en mesure d'effectuer le diagnostic de paludisme par PCR.

La stratégie thérapeutique est modifiée. De 2002 à 2007, les accès simples sont traités par l'association sulfadoxine-pyriméthamine. À partir de 2007, le traitement de première intention des accès simples utilise une combinaison thérapeutique à base d'artémisinine (CTA), l'association arthéméter-luméfantrine. Jusqu'en 2013, les accès graves sont traités par quinine intraveineuse. Depuis cette date, ils reçoivent un traitement par artésunate intraveineux. Dès que la voie orale est possible, un traitement complet de 3 jours par CTA est administré en relais.

Un système de surveillance est mis en place avec notification systématique aux autorités de santé par télécopie ou courriel de tous les cas de paludisme diagnostiqués par les médecins et les biologistes de l’île.

La lutte antivectorielle (LAV) est réorganisée et renforcée. L'objectif est désormais de 3 passages annuels dans chaque habitation de l’île pour des pulvérisations d'insecticide à effet rémanent. De 2012 à 2016, une campagne d’équipement de tous les foyers de l’île en moustiquaires à imprégnation durable (MID) a pris le relais des pulvérisations intradomiciliaires. Pour chaque cas notifié, une équipe mobile d'agents de la LAV intervient au domicile du malade. Une enquête épidémiologique est réalisée pour déterminer l'origine du cas et détecter d’éventuels cas secondaires. L'intérieur du domicile fait l'objet d'un traitement insecticide adulticide et est équipé en MID. Les gîtes larvaires productifs dans le voisinage de l'habitation sont recherchés et traités.

Ce nouveau programme de lutte contre le paludisme est-il efficace?

De janvier 2002 à décembre 2021, 4819 cas de paludisme acquis localement ont été notifiés à Mayotte. L'incidence annuelle a régulièrement diminué de 10,3 ‰ en 2002 (1649 cas) à moins de 0,01 ‰ en 2020 (2 cas). Depuis 2009, l'incidence des cas acquis localement est inférieure à 1 ‰. En 2013, l'OMS classe Mayotte parmi les territoires en phase d’élimination du paludisme. En 2021, aucun cas de paludisme acquis localement n'a été notifié sur l’île (Fig. 1).

**Figure 1:**
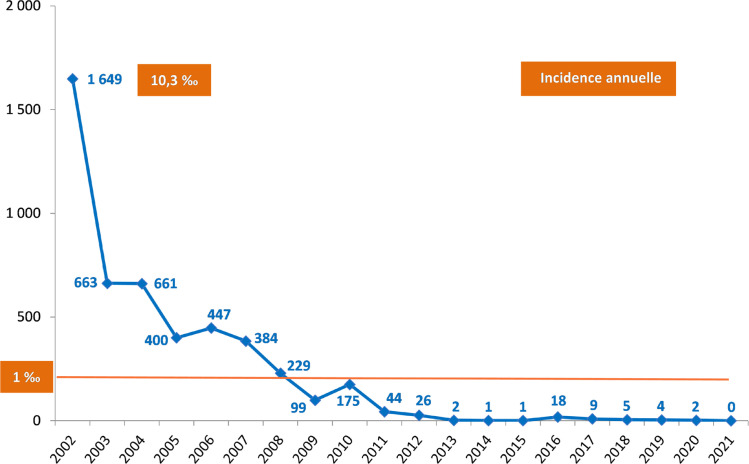
Nombre annuel et incidence des cas de paludisme acquis localement à Mayotte, 2002-2021 Annual number and incidence of locally acquired malaria cases in Mayotte, 2002-2021

Durant cette période 2002-2021, 1898 cas importés ont été observés. Ils proviennent principalement de l'Union des Comores (85,8%), de Madagascar (8,6%) et d'Afrique subsaharienne (5,6%).

À partir de 2017, le nombre annuel de cas acquis localement est inférieur à 10 et décroît régulièrement (9 cas en 2017, 5 en 2018, 4 en 2019 et 2 en 2020).

La répartition temporospatiale de ces rares cas acquis localement suggère qu'il s'agit de cas introduits et non de cas indigènes. L’étude du profil génotypique des souches plasmodiales des cas localement acquis observés de 2017 à 2020 (17 cas) confirme qu'il s'agit certainement de cas introduits en lien avec des cas importés de l'Union des Comores voisine.

Le dernier cas acquis localement a été notifié à Mayotte en juillet 2020. Eu égard aux caractéristiques du cycle des plasmodies, à l'absence d'immunité palustre de la population mahoraise et à la longévité des vecteurs anophéliens, le prochain cas acquis localement reporté en 2022 sur l’île ne pourra être qu'un cas introduit.

L'arrêt de la transmission indigène du paludisme semble donc être un fait acquis à Mayotte.

Pour l'OMS, une zone exempte de paludisme est une zone dans laquelle il n'y a pas de transmission locale continue du paludisme par le biais des moustiques et où le risque de contracter le paludisme se limite aux infections dues aux cas introduits.

Selon cette définition, l’île de Mayotte peut désormais être considérée comme une zone exempte de paludisme.

Toutefois ce territoire reste sous la menace d'une réintroduction *via* des cas importés des pays voisins. D'autant que la situation en Union des Comores est de nouveau préoccupante. Alors que l'incidence annuelle du paludisme y avait chuté à 2,3 ‰ en 2016 (1734 cas), on assiste depuis à une reprise de la transmission avec une incidence de 12,8 ‰ en 2021 (10 547 cas) [4]. À Mayotte, les vecteurs compétents sont toujours présents et les conditions environnementales permettant leur développement n'ont pas subi de modifications notables ces 20 dernières années.

Le programme de lutte contre le paludisme en vigueur actuellement à Mayotte doit rapidement être réorienté dans l'objectif de prévenir une réintroduction de la maladie. Au vu de l'environnement géographique de l’île, il est temps de mettre en œuvre une politique volontariste de coopération régionale en matière de lutte contre le paludisme.

## Remerciements

Les auteurs tiennent à remercier et à féliciter toutes les personnes impliquées dans la lutte quotidienne contre le paludisme à Mayotte: les professionnels de santé du Centre hospitalier de Mayotte et du secteur privé, les agents de l'ARS Mayotte et plus particulièrement ceux des départements de Surveillance et sécurité sanitaire et de la Lutte antivectorielle, ainsi que le personnel du Centre national de référence du paludisme à Marseille.

## Contributions Des Auteurs

Tous les auteurs ont contribué à la production et à l'analyse des données de cette étude. La première version du manuscrit a été rédigée par Jean-François Lepère et tous les auteurs ont commenté les versions suivantes du manuscrit. Tous les auteurs ont lu et approuvé le manuscrit final.

## Liens D'intérêts

Les auteurs déclarent ne pas avoir de liens d'intérêts.
